# Life, death, and 280 characters: a hashtag analysis of #AssistedSuicide on X (2015–2025)

**DOI:** 10.3389/fdgth.2026.1744855

**Published:** 2026-06-23

**Authors:** Elisa Opriessnig, Olena Litvinova, Zilin Ma, Yining Hua, ArunSundar MohanaSundaram, Maria Kletecka-Pulker, Atanas G. Atanasov, Thomas Wochele-Thoma

**Affiliations:** 1 Medical University of Graz, Graz, Austria; 2 Department of Management, Marketing and Quality Assurance in Pharmacy, National University of Pharmacy of the Ministry of Health of Ukraine, Kharkiv, Ukraine; 3 Ludwig Boltzmann Institute Digital Health and Patient Safety, Medical University of Vienna, Vienna, Austria; 4 Intelligent Interactive Systems Group, Harvard School of Engineering and Applied Sciences, Allston, MA, United States; 5 Department of Epidemiology, T.H. Chan School of Public Health, Harvard University, Boston, MA, United States; 6 Department of Biomedical Informatics, Harvard Medical School, Boston, MA, United States; 7 Department of General Internal Medicine, Brigham and Women’s Hospital and Harvard Medical School, Boston, MA, United States; 8 School of Pharmacy, Sathyabama Institute of Science and Technology, Chennai, Tamil Nadu, India; 9 Institute for Ethics and Law in Medicine, University of Vienna, Vienna, Austria; 10 Patient Safety & Digital Health (PaDiH) Group, Danube Private University, Fakultät Medizin/Zahnmedizin, Krems-Stein, Austria; 11 Department of Biochemistry, Saveetha Medical College and Hospital, Saveetha Institute of Medical and Technical Sciences, Chennai, Tamil Nadu, India; 12 Department of Biotechnology and Nutrigenomics, Institute of Genetics and Animal Biotechnology of the Polish Academy of Sciences, Magdalenka, Poland

**Keywords:** assisted suicide, death, life, mental health, palliative care, patient rights, social media

## Abstract

**Background:**

Population aging and the growing prevalence of chronic diseases pose significant challenges to the global healthcare system and highlight the relevance of issues related to the end-of-life stages. Social media is an important platform for discussing and exchanging views on these issues. This study aims to analyse public discourse on the X platform (formerly Twitter) between 2015 and 2025, based on the text content of posts (tweets) containing the hashtag #AssistedSuicide.

**Methods:**

A retrospective analysis was conducted on 105,130 posts containing #AssistedSuicide, published by 28,314 users on X between 2015 and 2025. Data were retrieved via Fedica (20 February 2025) and processed using Python 3. The study included quantitative content analysis, sentiment analysis, geographic distribution mapping, keyword and hashtag analysis, and assessment of legislative discourse and potential amplification patterns.

**Results:**

Most posts (99.1%) were in English. The highest activity was observed in the United Kingdom (39.48%), the United States (29.75%), Canada (13.94%), New Zealand (6.22%), and Australia (3.80%), accounting for 93.19% of all posts. Legislative-related content represented a substantial thematic category (38.7%) and also included issues concerning vulnerable populations. Analysis of frequently occurring words and hashtags shows that the discussion on #AssistedSuicide is multifaceted, polarized, and closely linked to legislative, medical, and social issues. The discourse is characterized by high sensitivity to political events and shifts in tone during periods of legislative activity.

**Conclusion:**

This study highlights the value of large-scale social media analysis for understanding public attitudes toward assisted suicide and identifying its ethical, social, and regulatory dimensions. Palliative care remains central to reducing suffering and preserving human dignity. The findings underscore the need for evidence-based policies that ensure protection of vulnerable groups, support healthcare professionals, and promote balanced end-of-life care frameworks.

## 1 Introduction

In recent decades, the global population has been steadily increasing and is projected by the United Nations to reach nearly 10.2 billion by 2080 (1). At the same time, population aging is occurring: the number of people over 60 is expected to double, and the number of individuals over 80 will rise from 146 million in 2020 to more than 426 million by 2050 (2). These trends are accompanied by a growing burden of chronic non-communicable diseases, including cancer, cardiovascular diseases, diabetes, metabolic syndrome, neurodegenerative disorders, and others (3).

Despite substantial medical advances in extending longevity, the quality of life in its final stages remains a critical challenge for humanity. This has led to increasing discussion of social responsibility for ensuring dignified conditions at the end of life. In recent decades, particular attention has been drawn to medical assistance in dying (MAiD) practices, including euthanasia and physician-assisted suicide, which are the subject of intense ethical, legal, and medical debate.

Assisted suicide, also referred to as physician-assisted suicide or medical assistance in dying (MAiD), is defined as the deliberate facilitation of another person's death, typically through the provision of prescribed lethal medications (4). The legal status of this practice varies considerably across jurisdictions (5). In a number of countries, including the Netherlands, Belgium, Luxembourg, Canada, and Switzerland, different forms of assisted dying are legally permitted, whereas in other states they remain prohibited or strictly restricted (6). In Austria, assisted suicide is permitted under strictly regulated conditions, whereas active euthanasia remains prohibited (7).

In recent years, patient autonomy has become a central theme in the discussion of assisted suicide. Researchers emphasise the need for a balance between autonomy and other ethical principles, including the protection of life and the interests of vulnerable groups. For instance, the concept of “relational autonomy” recognises that end-of-life decisions are formed in the context of social relationships and support systems, which requires ensuring awareness and effective communication (8).

In this context, social media are becoming important platforms for discussing ethically complex issues such as assisted suicide, while also serving as valuable tools for research. Social media analysis conducted by Jaye et al. revealed tensions in the interaction between citizens and state institutions in discussions on euthanasia and assisted dying (9). Wang et al. demonstrated that social media data can be effectively used to study palliative care and issues related to end-of-life care (10). Among social media platforms, X (formerly Twitter) is particularly significant due to its rapid real-time exchange of information and its ability to capture public sentiment through hashtag-based discussions (11). This makes it especially suitable for analysing public discourse on sensitive healthcare topics. This aligns with recent perspectives in digital health, which emphasize that social media analytics represent a promising tool for understanding public attitudes in healthcare (12, 13). However, despite this potential, researchers note that the existing literature on media coverage of euthanasia, including social media, is still limited, highlighting the need for further analysis of discussions on the X platform (14).

This study aims to analyse public discourse on the X platform (formerly Twitter) between 2015 and 2025, based on the text content of posts (tweets) containing the hashtag #AssistedSuicide.

By providing a comprehensive overview of the discourse on #AssistedSuicide on X over 10 years, this study contributes to a deeper understanding of how end-of-life issues in human life are framed, contested, and reimagined in the digital public sphere, which is an important step towards more informed and ethically grounded policy discussions.

## 2 Materials and methods

### 2.1 Study design

This retrospective observational study of publicly available social media data combined quantitative content analysis, sentiment analysis, and network-based indicators of user activity and content dissemination.

### 2.2 Characteristics of the Fedica platform and search strategy

For the analysis of public discourse, textual posts containing the hashtag #AssistedSuicide on the X platform (formerly Twitter) were used. The time frame covered 10 years, from 1 January 2015 to 1 January 2025.

Data collection was conducted using the Fedica analytics platform (https://fedica.com), a commercial web-based tool for monitoring and analysing social media activity (15, 16). The platform integrates with the official X API and provides access to aggregated public data, including post text, hashtags, engagement metrics. Fedica is not affiliated with the authors of this study, and its use is purely instrumental.

The search was conducted within the Fedica analytical environment rather than directly on the X platform. The query included filtering by the hashtag #AssistedSuicide and the specified time range. The data were exported in CSV format on 20 February 2025.

### 2.3 Data characteristics

The dataset includes 105,130 posts (including original posts, reposts, and replies) posted by 28,314 unique users, resulting in over 5.2 million impressions.

A key feature of platform X (formerly Twitter) is the limitation on message length (up to 280 characters), which encourages conciseness and stimulates the use of hashtags and symbolic forms of expression. At the same time, the built-in repost mechanism ensures instant and widespread dissemination of information, turning the platform into a significant indicator of public sentiment.

### 2.4 Inclusion and exclusion criteria

The inclusion criteria covered all publicly available textual posts containing the hashtag #AssistedSuicide within the specified time period. Posts without textual content (e.g., those containing only images, videos, or links without accompanying text), as well as posts consisting solely of a single hashtag without additional context, were excluded from the analysis, as they did not provide sufficient textual material for content analysis. The total number of excluded posts is not aggregated by the Fedica platform and therefore cannot be precisely quantified.

### 2.5 Activity analysis and content discourse analysis

Our analysis focuses on several key aspects of public discourse around #AssistedSuicide over 10 years, including the geographical distribution of posts, total impressions (views), the most frequently mentioned words and hashtags, and the most retweeted posts in the dataset. These metrics provide insight into both the reach and thematic focus of the #AssistedSuicide discussion across different regions and communities over time.

Engagement was defined as the aggregate of user interactions with posts (likes, reposts, replies, and quotes).

Posts in languages other than English were included in the overall quantitative analysis. However, given their negligible proportion (approximately 0.9% of the sample), linguistic analysis was conducted on English-language content. Posts were not translated, as their impact on the overall study results was considered insignificant.

### 2.6 Thematic categorization

The thematic categorization of the ten most frequently reposted posts was conducted using expert coding. Two independent researchers assigned thematic categories; in cases of disagreement, decisions were made through consensus. The method is based on qualitative content analysis approaches commonly used in studies of digital discourse (17, 18).

### 2.7 Analysis of legislative discourse

Legislative-initiative content was estimated with an explicit lexical code covering bills, laws, votes, parliamentary terms, courts, MAID, and named legislative hashtags. Theme summaries within the legislative subset were based on frequently repeated texts and high-volume lexical patterns.

### 2.8 Assessment of potential artificial amplification of discourse

To assess potential artificial amplification of discourse, reproducible concentration metrics were applied, including the number of unique users to posts, annual top-account shares, and duplicate-text prevalence.

### 2.9 Data preprocessing and cleaning

Posts with the hashtag #AssistedSuicide from 1 January 2015 to 1 January 2025 were saved in CSV format for further analysis. The dataset retrieved through Fedica includes all publicly available posts containing the hashtag #AssistedSuicide during the defined timeframe, ensuring comprehensive coverage of the discourse. Data processing was performed in Python 3 using the pandas library for data organization and analysis, as well as regular expressions for text cleaning. In the initial stage, URLs, reposts, user mentions, and special characters were removed, and the text was converted to lowercase and standardized for spaces. To highlight relevant content, a list of the most frequently mentioned words was compiled, from which common stop words (e.g., all, any, but, can, did, have, etc.) and domain terms not related to the topic of discourse (e.g., also, call, client, information, people, etc.) were manually excluded. A total of 244 words were removed, allowing us to focus on thematically relevant posts and identify key trends in discussions on #AssistedSuicide. The frequency distributions of keywords and hashtags were calculated, with the thirty most frequently occurring elements in each category being selected and exported for further analysis.

### 2.10 Sentiment analysis

The VADER (Valence Aware Dictionary and sEntiment Reasoner) algorithm, developed for social media analysis, was used to assess the sentiment of posts (19). Classification was based on the compound score: ≥0.05 indicated positive sentiment, ≤−0.05 indicated negative sentiment, and values between −0.05 and 0.05 were considered neutral. These threshold values are standard in automated sentiment analysis of social media data.

### 2.11 Ethical considerations of the study

The study exclusively included publicly accessible posts. No access was granted to personalized or deleted content, and no processing or provision of personal data was conducted. All procedures were carried out in accordance with the requirements of the General Data Protection Regulation (GDPR), which establishes standards for the protection of personal data in the European Union, including the principles of anonymisation, data minimisation, and restrictions on the processing of personal information.

This observational study analyzed publicly available posts from X (formerly Twitter) containing the hashtag #AssistedSuicide. Data collection was conducted through the Fedica analytics platform in strict accordance with X's Terms of Service and Developer Agreement. Additional safeguards were implemented: no direct quotations that could identify individual users were included, all results are presented exclusively in aggregate and anonymized form, and no attempts were made to contact users or access non-public information. All procedures complied with GDPR requirements and established ethical standards for observational social media research.

## 3 Results

### 3.1 General characteristics of the dataset

**Figure 1:**
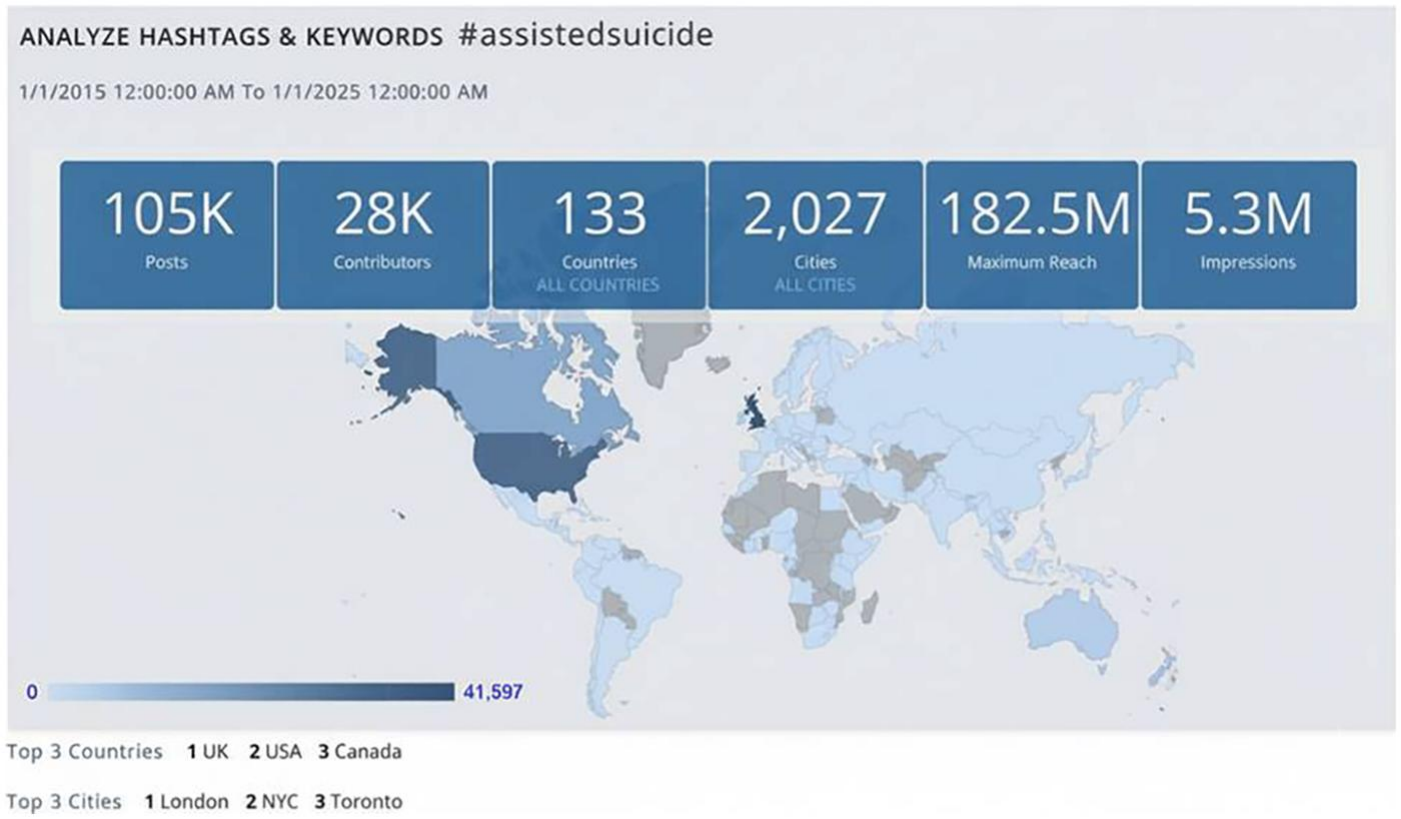
Overview of the characteristics of #AssistedSuicide posts (a summary-image directly derived from the Fedica interface).

Between January 1, 2015, and January 1, 2025, a total of 28,314 unique users on X (formerly Twitter) posted 105,130 posts containing the hashtag #AssistedSuicide. These posts generated over 5.2 million impressions, reflecting their high visibility and engagement (Figure 1). Interactions with the posts included 135,122 likes and 5,949 quotes, demonstrating that the discourse was actively discussed and widely shared.

Language analysis revealed that the vast majority of posts (99.1%) were in English, although some accounts posted content in other languages, including French, Spanish, Italian, German, Portuguese, Tamil, Dutch, Turkish, Catalan, Welsh, Bengali, and Japanese.

The dataset included 33,201 original posts, which were amplified by 65,688 reposts and mentioned in 6,261 replies.

### 3.2 Structural characteristics and activity dynamics of the dataset

The dataset contained 105,130 posts from 28,314 unique accounts, which yields a unique-user to post ratio of 0.269 and a mean of 3.71 posts per account. Duplicate normalized text represented 57.0% of all rows.

**Figure 2:**
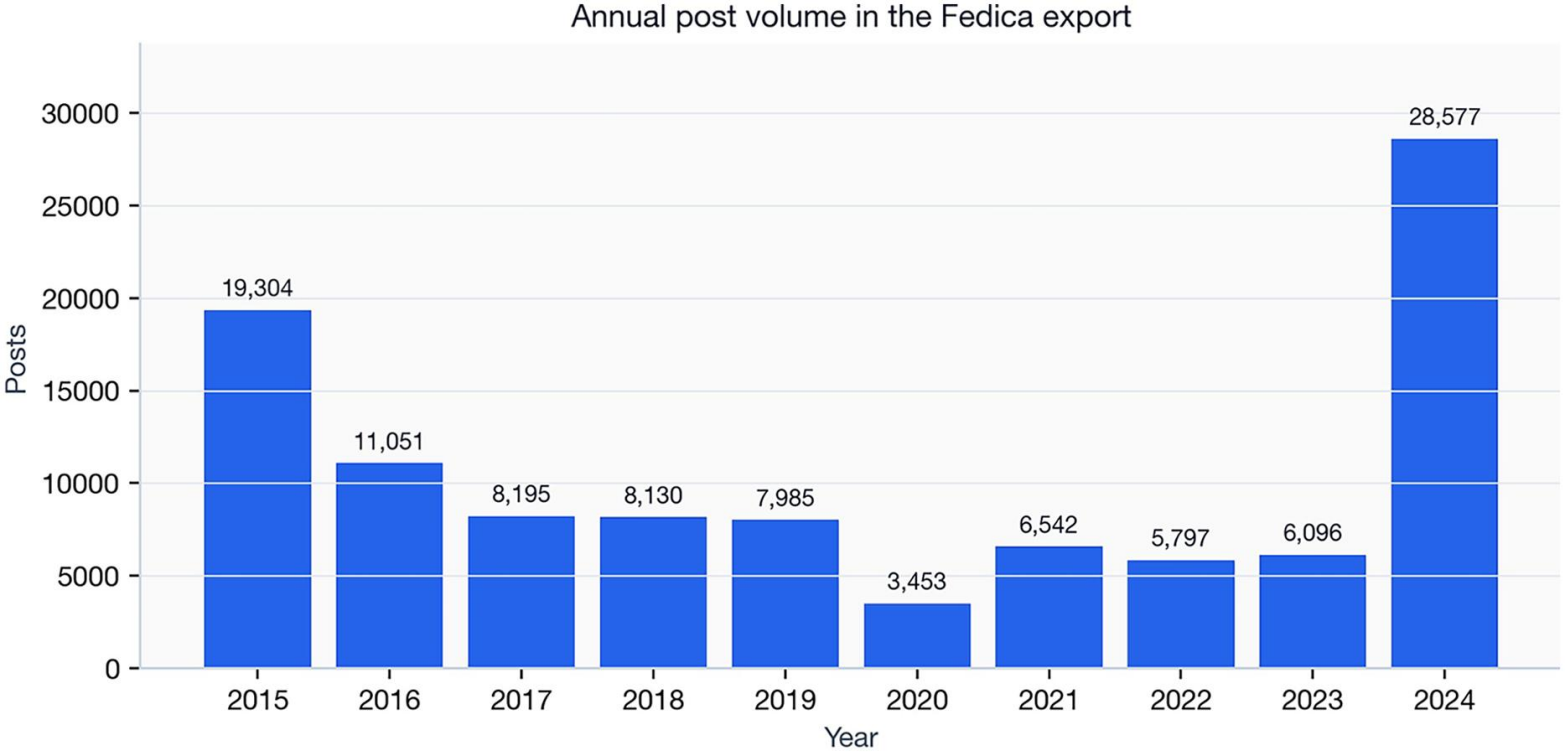
Annual post volume (January 1, 2015–December 31, 2024).

**Figure 3:**
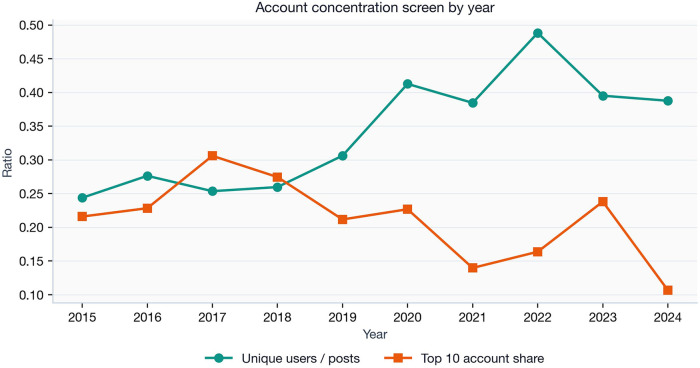
Account concentration by year. The green line represents the ratio of unique users to total posts, while the orange line shows the share of annual posts contributed by the 10 most active accounts*.*

No single account contributed more than 3.1% of the total dataset, while the 10 most active accounts accounted for 13.1% of all posts, indicating a moderate concentration of activity without dominance by individual users. The dynamics of posting activity show two pronounced peaks: in 2015 (19,304 posts) and in 2024 (28,577 posts), with a minimum in 2020 (3,453 posts) (Figure 2; Table 1). At the same time, the contribution of the most active accounts during peak periods varied. In 2015, the top 10 accounts accounted for 21.6% of posts, whereas in 2024 this figure dropped to 10.7%, indicating a broader distribution of user participation in the most recent surge of activity (Figure 3; Table 1).

**Table 1:** Annual activity and account concentration.

Year	Posts	Unique users	Users/posts ratio	Posts/user	Top 10 share (%)
2015	19,304	4,706	0.244	4.102	21.6
2016	11,051	3,054	0.276	3.619	22.8
2017	8,195	2,078	0.254	3.944	30.6
2018	8,130	2,111	0.260	3.851	27.5
2019	7,985	2,445	0.306	3.266	21.2
2020	3,453	1,425	0.413	2.423	22.7
2021	6,542	2,516	0.385	2.600	14.0
2022	5,797	2,831	0.488	2.048	16.4
2023	6,096	2,409	0.395	2.531	23.8
2024	28,577	11,080	0.388	2.579	10.7

### 3.3 Sentiment analysis

**Figure 4:**
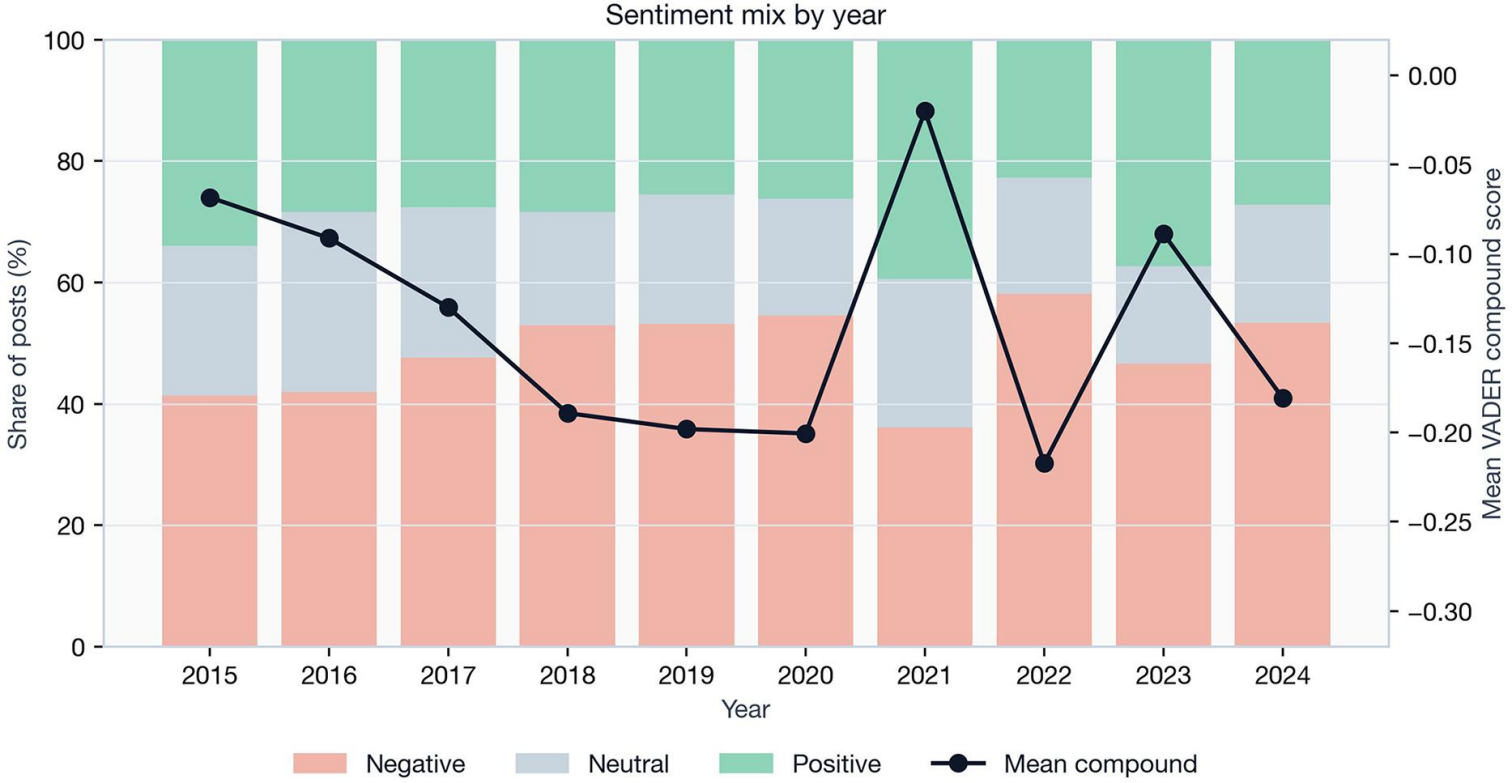
Sentiment distribution by year. The bars show the annual shares of negative, neutral, and positive posts. The black line represents the mean VADER compound score.

Overall sentiment was weighted toward negative framing. Across all posts, 48.3% were negative, 22.1% were neutral, and 29.6% were positive, with a mean compound score of −0.136. The least negative year was 2021, when the mean compound score was −0.020; the most negative years were 2022 and 2024. Posts coded as legislative were more negative than the remainder of the corpus (−0.168 vs. −0.116) (Figure 4; Table 2).

**Table 2:** Sentiment summary by analytic subset.

Subset	Posts	Mean compound	Negative (%)	Neutral (%)	Positive (%)
All posts	1,05,130	−0.136	48.3	22.1	29.6
Original posts	33,201	−0.142	48.4	22.6	29.0
Reposts	65,668	−0.128	48.0	22.0	30.1
Replies	6,261	−0.185	51.0	20.4	28.6
Legislative posts	40,712	−0.168	51.3	20.3	28.4
Non-legislative posts	64,418	−0.116	46.4	23.2	30.4

### 3.4 Legislative discourse

**Figure 5:**
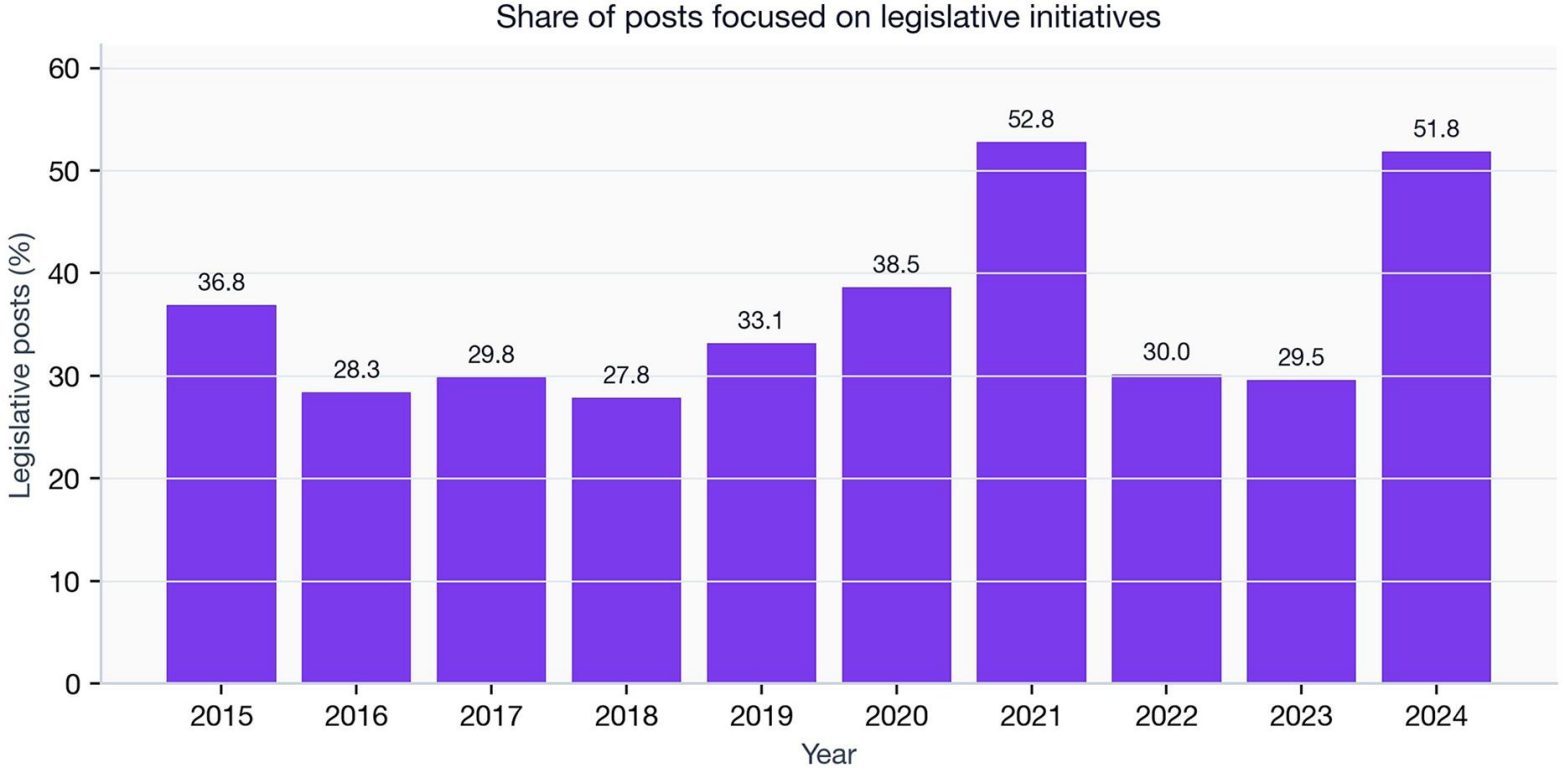
Share of annual posts coded as legislative initiative content using a transparent lexical rule.

Legislative initiatives accounted for 40,712 of 105,130 posts, or 38.7% of the full dataset. The share remained similar when the denominator was restricted to original posts only (35.4%) and when repeated texts were collapsed to unique normalized messages (34.9%). Legislative content was especially prominent in 2021 (52.8% of posts) and 2024 (51.8%), which indicates that legal and parliamentary developments were major drivers of the largest discussion spikes (Figure 5; Table 3).

**Table 3:** Share of content devoted to legislative initiatives.

Subset	Denominator	Legislative items	Share (%)
All posts	1,05,130	40,712	38.7
Original posts only	33,201	11,766	35.4
Unique normalized texts	45,243	15,773	34.9

The qualitative legislative screen showed one dominant theme and several secondary themes. The largest theme was “Bill process and voting”, present in 23,163 posts (56.9% of the legislative subset; non-exclusive coding). Secondary themes focused on disability rights and safeguards, Canadian MAiD precedent, and court challenges. Representative recurring messages, paraphrased to protect privacy, included claims that parliamentary votes were moving faster than disability-rights scrutiny, claims that Canada's Bill C-7 showed policy expansion after legalization, and claims that pending bills could increase pressure on disabled, older, or seriously ill people (Table 4).

**Table 4:** Non-exclusive qualitative themes in the legislative subset.

Theme	Posts	Legislative subset (%)	Interpretive note
Bill process and voting	23,163	56.9	Recurring posts tracked bill timing, parliamentary debate, and whip or free-vote decisions.
Disability rights and safeguards	9,503	23.3	Recurring posts framed disability rights, vulnerability, coercion, and safeguards as central objections.
Canada and MAID precedent	5,940	14.6	Recurring posts cited Canada and Bill C-7 as warning cases, often linked to MAID expansion.
Courts and legal challenges	2,397	5.9	Recurring posts discussed appeals, rulings, and judicial review as drivers of debate.
Palliative care and service impact	764	1.9	Recurring posts linked legal change to palliative-care shortages, cost pressure, or NHS strain.

### 3.5 The geographic distribution of posts

The geographic distribution of posts was diverse, covering numerous countries and regions (*n* = 133), highlighting the global relevance of the topic (Table 5). The highest number of #AssistedSuicide posts originated from the United Kingdom (39.48%), the United States (29.75%), Canada (13.94%), New Zealand (6.22%), and Australia (3.80), indicating strong public engagement with the issue of assisted suicide. Collectively, the top 5 countries accounted for approximately 93.19% of all posts. Posts from Australia and New Zealand together represented nearly 10%, emphasizing the significant contribution of this region alongside English-speaking countries in Europe and North America. The remaining posts were distributed across countries in Europe, Asia, and Africa, each contributing less than 3% of the total volume. The top three cities were London, New York, and Toronto.

**Table 5:** Top 5 countries by #AssistedSuicide activity.

Country	Total posts	Share, %
UK	41,597	39.48
USA	31,355	29.75
Canada	14,695	13.94
New Zealand	6,556	6.22
Australia	3,999	3.80
Other countries	7,173	6.81

### 3.6 Lexical and hashtag analysis

Systematization of content and engagement metrics of the 10 most reposted posts with #AssistedSuicide are presented in Table 6. An analysis of the content of the most reposted posts on X showed that the discourse on #AssistedSuicide is multifaceted and covers a wide range of topics: political and legal issues, ethical and social aspects, and social advocacy. Public debates include criticism of legislative initiatives and politicians' actions, discussion of coercion and vulnerability issues, and the protection of the interests and rights of people with disabilities. The highest level of engagement was recorded in posts that touched on political aspects and social advocacy, which indicates that the topic has become highly politicized in the public sphere.

**Table 6:** Systematization of content and engagement metrics of the 10 most reposted posts with #AssistedSuicide.

Rank	Engagement metrics	Post summary	Thematic category	Posting year
1.	Comments: 318; reposts: 4,361; likes: 9.8k; quotes: 283	The post criticizes the low attendance of lawmakers at debates on the rights of people with disabilities, despite many having recently voted in favor of legalizing assisted suicide.	Political and legal issues	2024
2.	Comments: 31; reposts: 583; likes: 1,966; quotes: 18	The post argues that instead of assisted suicide, support systems should be developed to improve the lives of people with disabilities.	Social advocacy	2024
3.	Comments: 147; reposts: 536; likes: 1,864; quotes: 87	The post describes a personal experience of being coerced into assisted suicide by a nurse.	Ethical and social aspects	2024
4.	Comments: 22; reposts: 425; likes: 771; quotes: 37	The author emphasizes that the UK Leadbeater bill to legalize assisted suicide has serious shortcomings and is not ready for safe implementation.	Political and legal issues	2024
5.	Comments: 73; reposts: 321; likes: 821; quotes: 36	Despite promises of strict safeguards, dozens of parliamentarians are pushing to expand access criteria for assisted suicide, which the author perceives as a premature change in the rules.	Political and legal issues	2021
6.	Comments: 23; reposts: 316; likes: 966; quotes: 29	A letter to the *Irish Times* conveys the author's view: before legalizing assisted suicide, it is necessary to ensure a dignified life and support for seriously ill people.	Political and legal issues	2024
7.	Comments: 49; reposts: 311; likes: 981; quotes: 16	The author urges not to pass the assisted suicide law in the UK, expressing concern for their autistic children who may be vulnerable to coercion.	Ethical and social aspects	2024
8.	Comments: 107; reposts: 294; likes: 822; quotes: 31	The author criticizes Canadian Prime Minister Trudeau for supporting assisted suicide in cases of mental disorders, highlighting the inconsistency with his image as a mental health advocate.	Political and legal issues	2024
9.	Comments: 16; reposts: 269; likes: 588; quotes: 24	The post warns of the risk that funds for palliative care may be redirected toward the cheaper option of assisted suicide.	Ethical and social aspects	2024
10.	Comments: 7; reposts: 263; likes: 414; quotes: 5	The post calls for disseminating statements from disability organizations opposing the legalization of assisted suicide under the Leadbeater bill.	Social advocacy	2024

**Figure 6:**
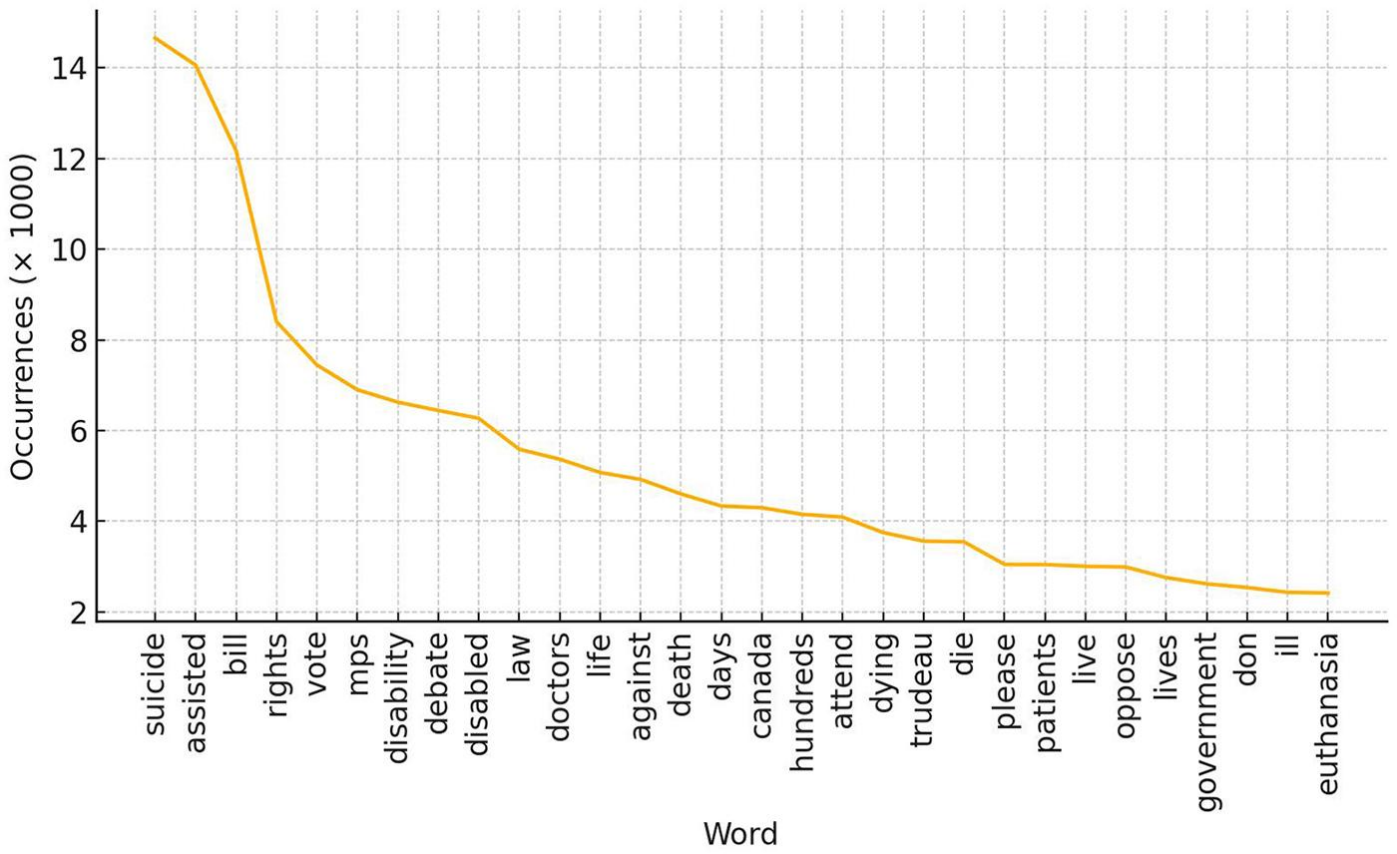
TOP-30 most frequently mentioned words occurring together with the hashtag #AssistedSuicide.

**Figure 7:**
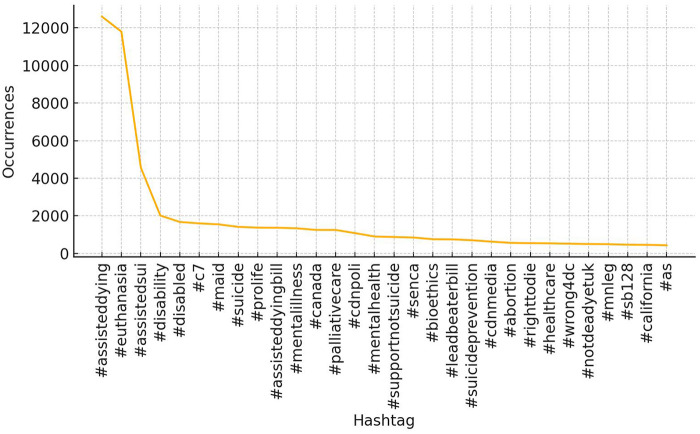
TOP-30 most frequently mentioned hashtags occurring together with the #AssistedSuicide.

One of the informative indicators of the X analysis is the identification of the most frequently mentioned words and hashtags associated with #AssistedSuicide. The results of this analysis are presented in Figures 6, 7. As shown in Figure 6, the most frequently mentioned words were “suicide” (14,653) and “assisted” (14,057), highlighting the centrality of the topic of assisted suicide in public discourse. The third most common word was “bill” (12,169), reflecting attention to legislative initiatives related to this issue. Additionally, discussions prominently featured terms associated with the rights of people with disabilities (“rights,” “disability,” “disabled”), medicine and bioethics (“doctors,” “death,” “dying,” “patients,” “euthanasia”), as well as the political context (“Canada,” “Trudeau,” “government”). This underscores the multi-layered nature of the discourse, intertwining legal, social, medical, and political aspects of the issue. Beyond the 30 most frequently occurring words, the discourse also includes less common but noteworthy mentions of “terminal sedation,” “cancer,” “oncology,” and “oncologist,” highlighting the relevance of these aspects in end-of-life care.

Analysis of the data presented in Figure 7 shows that the most frequent hashtags are #assisteddying (12,596) and #euthanasia (11,795), reflecting the main topic of discussion. The hashtag #MAiD (1,545 mentions), referring to medical assistance in dying, is mainly used in the context of Canadian legislation.

The hashtag analysis highlights the diversity of positions within the discourse. The use of tags such as #prolife, #supportnotsuicide, and #righttodie reflects a polarization of views and the presence of opposing argumentative lines. At the same time, hashtags such as #mentalillness and #palliativecare are frequently employed, indicating the salience of medical and ethical aspects in the public debate. The geographical and political aspects of the discussion are reflected in the hashtags #canada, #cdnpoli, and #c7, which refer to Canadian Bill C-7, emphasizing that changes in legislation have become a key factor in public debate.

The combined use of these hashtags demonstrates that the debate on assisted suicide is closely intertwined with discussions on mental health, the rights of people with disabilities, and the quality of palliative care, particularly in the context of oncology.

Overall, the analysis of hashtags confirms the results obtained from the analysis of words and demonstrates that the discussion has a clear thematic structure, expresses polarization of opinions, and has a close connection to specific legislative and political events.

## 4 Discussion

This study presents an analysis of digital discourse on the hashtag #AssistedSuicide on the X platform over 10 years and demonstrates that discussions of euthanasia on social media are strongly event-driven, politicized, and polarized in nature. The results provide valuable insights into how social media users engage with ethically complex health issues and how this discourse is shaped by geographical and thematic characteristics.

### 4.1 General characteristics, structure, and temporal dynamics of the public discourse on the X platform

The volume of activity, 105,130 posts from 28,314 users, generating over 5.2 million views, reflects sustained and widespread interest in the topic of assisted suicide over a decade. The diversity of participants and the scale of the audience indicate that social media, and X in particular, have become a significant platform for sharing personal experiences, discussing policy, and expressing positions on the legal, social and ethical aspects of end-of-life decisions.

The discourse structure is characterised by a predominance of reposts (≈62%) and a high proportion of duplicated content (57%), indicating that mechanisms for the dissemination and amplification of information take precedence over the creation of original posts. The results obtained indicate a high degree of information circulation. They may point to signs of structural clustering within the discourse, whereby certain narratives—including criticism of legalisation and an emphasis on risks to vulnerable groups—demonstrate heightened visibility and persistence within the information landscape.

The dynamics of publication activity are markedly uneven and event-driven. The pronounced peak in 2015 is likely linked to the intensification of parliamentary debates on assisted dying, primarily in the UK, which also contributes significantly to the overall dataset. The significant decline in activity in 2020 appears to be due to the COVID-19 pandemic, which shifted public attention towards emergency healthcare issues and temporarily reduced the intensity of discussion on bioethical topics. The most significant increase in activity in 2024 coincides with a new wave of international legislative initiatives, including the expansion of medical assistance in dying (MAiD) in Canada and intensified political debates in other countries.

The patterns observed suggest a possible link between the dynamics of the discourse and the legislative agenda, as evidenced by an increase in the proportion of content relating to legal issues (exceeding 50% at certain times), as well as a shift in the tone of the posts. Taken together, this suggests that the discussion of assisted suicide on social media is reactive in nature, reflecting the sensitivity of public attention to significant political and legal events. The findings are consistent with Jaye et al., who also report an association between social media discourse on euthanasia/assisted dying and political and legal developments, as well as its ambivalent nature (9).

The results of lexical and hashtag analysis demonstrate a marked polarisation of the discussion, reflected in the simultaneous use of hashtags with opposing meanings, such as #prolife and #righttodie. This points to the coexistence of competing moral and ethical frameworks, within which euthanasia is interpreted either as the realisation of patient autonomy or as a potential threat to vulnerable groups. Such polarisation may contribute to the increasingly confrontational nature of the discourse and complicate the achievement of consensus in public and political discussions.

The prevalence of a negative tone in the discourse (48.3%), as well as its intensification in the context of legislative debates, indicates the dominance of a framework focused on risks and potential threats. In particular, discussions relating to legislative initiatives are often accompanied by expressions of concern regarding potential abuses, pressure on vulnerable groups, and inadequate protection for patients. This may contribute to the formation of a more cautious or critical public attitude towards the expansion of assisted suicide practices. At the same time, Riley et al. emphasize that user reactions to euthanasia narratives on Twitter are characterized by emotional engagement and a plurality of perspectives, including personal experiences and diverse interpretations of end-of-life practices (20).

An analysis of participation patterns reveals that no individual users exert significant dominance. No single account accounted for more than 3.1% of the total volume of posts, while the ten most active accounts together accounted for 13.1%. At the same time, during periods of heightened activity, there is a decrease in the concentration of posts among the most active participants, indicating a broadening of engagement and a reduction in the concentration of discourse. This is consistent with the findings of Lalancette et al., according to which ordinary users actively participate in discussions on medical assistance in dying, extending public debate beyond expert and institutional circles (21).

Changes specific to the platform (including the transition from Twitter to X and modifications to the moderation policy after 2022) may have influenced the visibility and dissemination of content, which should be taken into account when interpreting the temporal dynamics of the discourse.

### 4.2 Geography and legislative framework of discourse

A geographical analysis has shown that the highest level of activity under the hashtag #AssistedSuicide is concentrated in English-speaking countries—primarily the United Kingdom, the United States, Canada, and New Zealand. This indicates a close link between the intensity of digital discourse and the national legal context, and confirms that social media serves a dual function—as a tool for responding to legislative initiatives and as a platform for civic engagement.

The United Kingdom, where euthanasia and assisted suicide remain illegal, contributed most to the discussion. Despite this, the high level of activity reflects ongoing public and professional debate, which is consistent with the literature on significant ethical and clinical discussion on this issue (22). This confirms the finding in our analysis that the intensity of discourse tends to be high precisely where legalisation is absent, particularly in the context of active political debate.

The United States ranks second in terms of activity, reflecting a complex and fragmented legislative landscape. Partial legalisation at the state level is accompanied by persistently limited public awareness and heterogeneity in public opinion (23). This is consistent with our findings, which demonstrate a high degree of polarisation in the discourse and the coexistence of opposing normative positions.

The Canadian experience serves as a key example of the impact of legalisation on public digital discourse. According to the literature, following the introduction of medical assistance in dying (MAiD), there has been a steady increase in the number of cases, as well as an intensification of ethical and organisational debates (24–27). Further research points to specific socio-demographic characteristics of MAiD recipients and the persistent burden of physical and psychological suffering, despite access to palliative care (28). These observations are consistent with our findings, which demonstrate an increase in the proportion of legislative content and a rise in negative sentiment during periods of regulatory expansion, as well as frequent references to vulnerable groups. Taken together, this suggests that legislative liberalisation may simultaneously intensify public engagement and polarise digital discourse.

In New Zealand, the legalisation of medical assistance in dying in 2021 followed a period of vigorous public debate and revealed discrepancies between public expectations and policy (9). This confirms the event-driven nature of the discourse identified in our study, whereby online activity increases during periods of legislative change.

A comparative analysis of other jurisdictions, including Belgium, Switzerland, the Netherlands, and certain US states, shows that the legalisation of euthanasia is accompanied by an increase in the number of cases and the development of regulatory mechanisms, but also leads to the emergence of new ethical and organisational challenges (29–32). Despite differences in national models, these findings are consistent with our results, demonstrating that digital discourse is characterised by contextual dependence, polarisation, and heightened attention to issues of vulnerability and social protection.

Beyond legal and geographic patterns, understanding the demographic and clinical characteristics of individuals involved in assisted suicide is also important for interpreting public discourse. The literature on the characteristics of patients receiving MAiD or assisted dying indicates a predominance of individuals in older age groups (mainly 75–80 years old) with oncological and other progressive chronic diseases (25, 30, 32). Several studies also report a higher proportion of men, patients living at home, and individuals who are married, as well as, in some cases, those with relatively higher socioeconomic status (28, 32). Despite differences across jurisdictions, a key common factor remains the presence of severe physical and psychological suffering (28).

The findings thus confirm that the digital discourse on assisted suicide is closely linked to national legal frameworks and intensifies during periods of legislative change. Platform X serves as a space for transnational exchange of views, within which not only legal models are discussed, but also their potential impact on vulnerable groups, including older people and people with disabilities.

### 4.3 Palliative care in public discourse on the X platform concerning assisted suicide

Online discourse on assisted suicide and euthanasia actively emphasizes the need to prioritize the development of palliative care. Users note that before discussing the legalization of EAS (euthanasia and assisted suicide), people with serious illnesses should have decent living conditions, psychological support, and access to quality care. This emphasis is reflected in key hashtags and words (#palliativecare, #supportnotsuicide, #disability, #notdeadyetuk), where investment in care is contrasted with hastening death. Palliative care is presented as the primary measure aimed at alleviating suffering and protecting human dignity, while euthanasia or assisted suicide is seen as an extreme and strictly regulated alternative. This is consistent with the findings of Wang et al., who reported a growing interest in palliative care research within social media discussions (10).

Our findings show that during periods of intensified legislative debate and negative rhetoric, palliative care becomes an important topic, particularly in discussions concerning vulnerable groups and the risks of inadequate care. This highlights its central role in shaping critical narratives in the digital sphere.

These observations are consistent with international research, in which palliative care is defined as a comprehensive approach aimed at improving the quality of life of patients and their families (33). Various jurisdictions emphasise the need to combine the regulation of assisted suicide with the provision of accessible, high-quality palliative care, as well as strict ethical and clinical standards (34, 35). Systematic reviews demonstrate that the relationship between palliative care and euthanasia can be both complementary and conflicting in nature (36).

An additional aspect of the discourse is terminal sedation as a clinical alternative aimed at relieving unbearable suffering without the intention of hastening death (37). International guidelines emphasise the need to adhere to the principles of proportionality, patient autonomy and clinical justification when applying it (38). Empirical data, however, indicate variability in practice across countries and a broadening of indications (39).

Overall, palliative care is a key element of the digital and clinical discourse on assisted suicide. It plays a central role in alleviating severe physical and psycho-existential suffering, emphasizing its importance as a fundamental approach and providing a scientific rationale for prioritising investment in high-quality end-of-life care.

### 4.4 Clinical aspects of public discourse on the X platform concerning assisted suicide: oncology and end-of-life care

Oncological topics regularly feature in the digital discourse surrounding #AssistedSuicide, reflecting their significance in the context of discussions. Our data show that references to cancer and oncological diseases are consistently present in the dataset, but do not dominate, giving way to broader issues related to the rights of people with disabilities, the accessibility of palliative care, and the regulatory and ethical aspects of legalisation.

In clinical practice, oncological diseases remain one of the most common indications for the use of euthanasia and assisted suicide in several countries. This is due to the high burden of physical and psycho-emotional suffering characteristic of the terminal stages of the disease, which necessitates comprehensive medical and psychosocial support.

The literature emphasizes that approaches to managing such patients vary considerably across jurisdictions and require a balance between patient autonomy and compliance with ethical and legal standards in end-of-life care (40).

A comparison of these findings with the results of our analysis shows that oncology represents an important but embedded component of a broader digital discourse in which medical, legal, and social dimensions are intertwined. Thus, cancer plays a significant but non-dominant role in discussions of assisted dying, reflecting a shift in public attention away from purely clinical factors toward more complex socio-ethical issues.

### 4.5 Assisted suicide in the context of mental health and vulnerability

The prominent presence in public discourse of terms relating to disability and mental health, as well as their frequent mention in the context of legislative initiatives, indicates that public debates on euthanasia are closely linked to issues of social vulnerability. Users actively express concern that expanding access to assisted suicide may have a disproportionate impact on people with disabilities, chronic illnesses, or mental health conditions. This highlights the need to take social determinants of health into account when developing relevant policies.

The right to assisted suicide for people with mental disorders (MAiD-NT, medical assistance in dying—not terminal) remains the subject of intense ethical, legal, and public debate. In the discourse under study, the hashtags #mentalillness, #assistedsui, and #supportnotsuicide reflect criticism of Canadian Prime Minister J. Trudeau for supporting the expansion of MAiD, as well as users' concerns about the risks to vulnerable groups, including children with autism. Despite promises of strict restrictions, the discussion of expanding the criteria is perceived as premature.

The literature confirms the need for a cautious approach to assisted suicide in cases of mental disorders. A systematic review conducted by Calati et al. notes an increase in requests for euthanasia in cases of mental disorders and emphasises the need for a clear distinction from suicidal behaviour (41). Grassi et al. point out that MAiD-NT is legalised to a limited extent (the Netherlands, Belgium, Luxembourg) and requires enhanced clinical assessment and patient safeguards (42). Colleran et al. emphasise that the legalisation of euthanasia in the Netherlands, Belgium, and Canada is accompanied by the introduction of multi-level safeguards, including psychiatric and palliative care, aimed at preventing coercion (43). Downie et al. further highlight the influence of socio-economic factors, which exacerbate the risk of unequal access and the “slippery slope” (44).

In Canada, the right to MAiD for individuals whose sole medical condition is a mental disorder has been postponed until 17 March 2027; restrictions are in place to protect vulnerable groups (45).

Overall, a comparison of data from social media and the academic literature shows that extending the right to euthanasia to people with mental health conditions remains one of the most complex and contentious areas. The findings highlight the need for rigorous assessment procedures, consideration of the social determinants of health, and enhanced protection for vulnerable groups when developing relevant policies.

### 4.6 Ethical and professional challenges for healthcare professionals in EAS

Analysis of publications using the hashtag #AssistedSuicide revealed that online discourse focuses on the roles of “doctors” and “patients,” as well as the concepts of “life” and “death.” This reflects a broad interest in the ethical and professional aspects of healthcare professionals' involvement in assisted suicide. Literature data confirm that physicians' and nurses' engagement in end-of-life care, including assisted suicide, is associated with a significant moral and emotional burden.

Our data suggest that healthcare professionals' involvement in euthanasia and assisted suicide is perceived as an ethically complex and emotionally taxing practice. This is consistent with the literature, which demonstrates significant moral pressure and stress among professionals: according to a review, between 15% and 50% of doctors experience marked emotional distress, although some report professional satisfaction linked to respecting patient autonomy (46).

An analysis of 30 studies (2017–2023) conducted by Pinto et al. showed that doctors' involvement in euthanasia or assisted suicide is accompanied by moral stress, increased workload, and burnout. 45.8%–80% of participants reported negative consequences, yet only a few sought help (47). Similar dilemmas are also characteristic of nursing staff, whose attitudes vary depending on personal, professional, and cultural factors (48).

As access to euthanasia expands, the importance of clinical training and emotional resilience among healthcare professionals is growing, as is the need for interdisciplinary collaboration, including the integration of palliative care (49). A particular role is assigned to psychiatrists, who are regarded as key participants in the assessment of complex cases. The literature emphasises the importance of psychiatric assessment for analysing the patient's motivation and ruling out factors associated with mental disorders (50).

Overall, a comparison of data from social media and the scientific literature shows that the involvement of healthcare professionals in EAS presents significant ethical and professional challenges. The findings highlight the need for institutional support, systematic training, and the development of psychological support mechanisms for healthcare professionals.

### 4.7 The broader ethical, cultural and social aspects of the public discourse on the X platform concerning assisted suicide

Public discourse on assisted suicide on platform X (#assisteddying, #assistedsui, #maid, #assisteddyingbill, suicide, assisted, die, patients, doctors, disability, disabled, rights, palliative care, healthcare, bioethics, government) in society has focused on issues of regulation and legal restrictions. Posts emphasize the need for transparent procedures and strict controls.

Our findings show that ethical debates in the digital sphere are characterised by a predominantly negative tone (48.3%, mean compound score −0.136), which intensifies during periods of active legislative debate (−0.168 vs. −0.116). This indicates the dominance of risk-oriented moral narratives, particularly in the context of expanding access criteria and legal regulation. At the same time, the high proportion of legislative content (up to 52.8% during peak periods) confirms the close link between moral debates and political and legal events.

Assisted suicide remains one of the most controversial topics at the intersection of law, medicine, religion, and individual rights (51, 52). The online discourse analyzed in this study provides a valuable source for assessing public opinion, moral judgments, and advocacy related to end-of-life decisions. For policymakers and public health professionals, such data are particularly important given the ongoing discussions and legislative reviews in this area (53). In addition, social media analysis reveals public concerns about access, equality, autonomy, and safeguards, aspects that are central to ethical discussions but often underrepresented in official policy debates (54). Yu et al. also demonstrate, based on YouTube comments, that such discourses, including discussions of so-called “suicide travel,” are shaped by legal, religious, and humanitarian arguments, including issues of human rights and perceptions of the dying process (55).

Literature data confirm the complexity of ethical, legal, and social aspects of euthanasia and assisted dying. Fontalis et al. emphasize that assisted dying is ethically controversial and regulated differently in different countries, sparking debates about the balance between patient autonomy and the right to life (4). The review emphasizes that many medical professionals are not prepared to make such decisions.

Cultural and religious factors have a significant influence on attitudes towards euthanasia. Studies show that the degree of religiosity correlates with a more critical view of euthanasia, and in several traditions, it is expressly prohibited or strictly restricted (56–58). Comparative data also point to variations in practices between countries, driven not only by legislation but also by cultural and institutional characteristics (59, 60).

The desire to hasten death in people with incurable diseases varies in intensity and is associated with depression, pain, functional limitations, loss of meaning in life, feelings of being a burden, and reduced quality of life, highlighting the need for clinical strategies to identify such cases (61). It has also been noted that the debate surrounding euthanasia for non-terminally ill patients and older adults raises important ethical and social concerns, as well as the need to involve various stakeholders in policy-making (62). In particular, the literature also points to a potential risk of a copycat effect associated with the nature of media coverage of euthanasia, including social media (14).

Contemporary philosophical and legal debates show that there is no consensus on the issues of assisted suicide and euthanasia. Supporters emphasize the humanitarian aspect, respect for patients' rights, and the role of legislation in controlling the procedure, while opponents point to the risks of hasty decisions, religious prohibitions, the possibility of abuse, and the duty of doctors to preserve life. The main objections relate to medical ethics, the likelihood of diagnostic errors, the slowdown in the development of new treatments, and exceptional cases of rare recovery. At the same time, states must provide alternative measures of assistance, including access to quality palliative care that can alleviate suffering and improve the quality of life of patients in the final stages of life.

Given the diversity of opinions, cultural and religious differences, and legal restrictions, the debate on assisted suicide must continue. Gradual and reasoned discussion in scientific, medical, and public contexts will allow for the development of coordinated approaches and bring us closer to a future consensus.

This study offers the first large-scale investigation of discourse on assisted suicide on platform X, relying on a unique 10-year dataset (2015–2025) comprising over 105,000 posts from 28,314 users. The use of modern tools (Fedica, Python) ensured the transparency and reproducibility of the analysis. A global perspective revealed the dependence of the intensity of online discussions on national legislative contexts. The data obtained has practical value, demonstrating key areas of social polarization and pointing to the potential of social media analytics for the development of ethically sound health care policies.

Unlike traditional sociological research based on surveys, social media analysis makes it possible to capture users' spontaneous, unprompted reactions to current events. This makes such data particularly valuable for understanding the dynamics of public opinion in real time, especially in the context of rapidly evolving legislative processes.

This study has several limitations. The use of a single English-language hashtag (#AssistedSuicide) introduces a degree of linguistic and geographical bias into the sample. Despite the global relevance of the topic, the analysis primarily reflects the English-speaking segment of digital discourse on the X platform, resulting in a relative overrepresentation of Anglosphere countries (the United States, the United Kingdom, Canada, and New Zealand). This reduces the representation of regions with active but non-English-language debates on euthanasia (e.g., the Netherlands, Belgium, Luxembourg, Switzerland, Spain, and countries in Latin America), thereby limiting the generalisability of the findings.

The analysis was restricted exclusively to textual data from the X platform. The exclusion of multimodal content (images, videos, memes, hyperlinks) limits the completeness of the reconstructed digital discourse, given the increasing role of visual and hybrid forms of communication in shaping public opinion. The use of a single hashtag as a data retrieval criterion reduces sensitivity to terminological variation and semantic equivalents (e.g., #euthanasia, #MAiD, #righttodie).

The data were collected using a single analytical platform (Fedica), which implies dependence on its aggregation algorithms and the limitations of access to the X API.

At the same time, these limitations are typical of social media-based research and do not undermine the internal validity of the identified thematic, temporal, and sentiment patterns. Future research could improve external validity by expanding language coverage, incorporating multimodal data, and applying a multi-platform analytical approach.

## 5 Conclusions

Social media, particularly platform X, has become an important venue for transnational discussion of end-of-life issues. An analysis of posts with the hashtag #AssistedSuicide between 2015 and 2025 shows that digital discourse reflects legal changes, as well as public concerns about vulnerable groups, including older people and people with disabilities.

The public discourse on the X platform concerning assisted suicide is closely linked to the legislative agenda. Almost 40% of posts focus on legal initiatives, indicating that the discourse is highly politicised and dependent on regulation changes. During periods of political initiatives and reforms, user activity increases and the tone of posts becomes more negative. This indicates that public opinion is highly sensitive to changes in the regulation of this issue.

The results demonstrate a high level of polarisation of opinion and the overlap of the discussion with topics such as human rights, mental health, disability, cancer, medical professionals, and palliative care. The geographical reach and user engagement metrics confirm the English-speaking global nature of the discussions, despite differences in legislation and cultural values.

The results highlight the need for systematic monitoring of social media as an indicator of public sentiment in the field of healthcare and bioethics. With the growth of digital participation among citizens, these platforms can be used not only by researchers but also by legislators, medical organizations, and human rights groups to identify vulnerable groups, assess public reaction to draft legislation, and develop strategies for informing the public. These insights are valuable, but they remain limited to one platform, one hashtag, and English-language content; future studies should expand across multiple platforms and languages to validate and extend our findings. In the long term, integrating social media analysis into the decision-making process will increase the transparency and quality of public and medical discussions, ensuring a more balanced and ethically sound approach to end-of-life issues.

## Data Availability

The original contributions presented in the study are included in the article, further inquiries can be directed to the corresponding authors.
